# Multi-step screening of neoantigens’ HLA- and TCR-interfaces improves prediction of survival

**DOI:** 10.1038/s41598-021-89016-7

**Published:** 2021-05-11

**Authors:** Guilhem Richard, Anne S. De Groot, Gary D. Steinberg, Tzintzuni I. Garcia, Alec Kacew, Matthew Ardito, William D. Martin, Gad Berdugo, Michael F. Princiotta, Arjun V. Balar, Randy F. Sweis

**Affiliations:** 1 EpiVax Therapeutics, Inc, 188 Valley Street, Suite 424, Providence, RI 02909 USA; 2 EpiVax, Inc, Providence, RI USA; 3 University of Georgia, Athens, GA USA; 4 NYU Langone Health, New York, NY USA; 5 University of Chicago, Chicago, IL USA; 6 Present Address: Editas Medicine, Cambridge, MA USA

**Keywords:** Bladder cancer, Computational platforms and environments, Bladder cancer, Prognostic markers, Tumour immunology, T cells, MHC

## Abstract

Improvement of risk stratification through prognostic biomarkers may enhance the personalization of cancer patient monitoring and treatment. We used Ancer, an immunoinformatic CD8, CD4, and regulatory T cell neoepitope screening system, to perform an advanced neoantigen analysis of genomic data derived from the urothelial cancer cohort of The Cancer Genome Atlas. Ancer demonstrated improved prognostic stratification and five-year survival prediction compared to standard analyses using tumor mutational burden or neoepitope identification using NetMHCpan and NetMHCIIpan. The superiority of Ancer, shown in both univariate and multivariate survival analyses, is attributed to the removal of neoepitopes that do not contribute to tumor immunogenicity based on their homology with self-epitopes. This analysis suggests that the presence of a higher number of unique, non-self CD8- and CD4-neoepitopes contributes to cancer survival, and that prospectively defining these neoepitopes using Ancer is a novel prognostic or predictive biomarker.

## Introduction

Understanding mechanisms of cancer progression and identifying patients at high risk for recurrence are pivotal to the personalization of cancer care. Improvements in DNA sequencing techniques combined with cost reductions have enabled the routine mapping of the tumor genome and improved our mechanistic understanding of cancer progression and patients’ survival. Tumor mutational burden (TMB) has arisen as a potential cancer prognosis biomarker in numerous tumor types^1,2^. Higher TMB has been associated with improved survival, highlighting the link between immune recognition of tumor neoantigens and favorable clinical outcomes. In solid tumors, the generation of an adaptive anti-tumor immune response requires a complex coordination of events ultimately dependent on cross-presentation of tumor-associated or tumor-specific antigens and cytotoxic T lymphocyte recognition of antigenic peptides presented on class I major histocompatibility complex (MHC), or human leukocyte antigen (HLA), of tumor cells. Attention has shifted to neoantigens generated by somatic mutations, since their recognition by the immune system is less impacted by central tolerance mechanisms, and as they are the targets of effector CD8+ T cells post checkpoint blockade therapy^3,4^. An increase in the absolute quantity of tumor mutations and neoantigens has been associated with favorable response to checkpoint immunotherapy and has led to a tumor histology-agnostic regulatory drug approval^2^.

Importantly, the quality of each potential antigen may critically affect the likelihood of mounting an effector T cell anti-tumor immune response^5–7^. Thus, an individual patient’s prognosis is likely to be influenced not only by the quantity of neoantigens, but by the presence of neoantigens that are most likely to result in an effective anti-tumor immune response. Optimal neoantigens may be defined by several factors including the level of neoantigen gene expression, the processing of peptide fragments, the binding affinity of neoantigen peptide fragments to HLA, successful presentation on the surface of the cell, and the phenotype of the immune cell that responds to the neoantigen. The exclusion of potential regulatory T cell (Treg) epitopes in the tumor mutanome has been one focus of our cancer vaccine development program^8^. Other groups have also reported that tolerizing epitopes may arise during the mutational process, and that these suppressive epitopes have a deleterious effect on cancer vaccine efficacy^9^.

Because cancers arise from self, neoepitopes bound to HLA molecules must be sufficiently different from endogenous peptides in order to be recognized as non-self by the patient's existing T cell repertoire. A novel epitope where none previously existed is most readily identified as non-self, however, mutations that change the T cell receptor (TCR) facing portion of an existing epitope can also influence the immune response to an antigen. TCR-facing amino acids that contain sequences resembling the unaltered human genome have been shown to modulate immunity by activating a tolerizing response. This has been observed in the context of infectious disease antigens, where pathogen-derived epitopes presenting a TCR "face" homologous to self-derived epitopes elicited CD4^+^CD25^+^FoxP3^+^ (regulatory) T cell (Treg) responses^10,11^, which in turn led to the suppression of effector immune responses against co-administered epitopes^11^. Removal of self-like epitopes from vaccine formulations has shown to increase immunogenicity of H7N9 influenza and CT26 vaccines^8,12^ and protection against lethal H7N9 challenges^13^. Preliminary work from our group in oncology suggests these "self-like" inhibitory neoepitopes also exist in mutated antigens derived from the murine colon carcinoma CT26 cell line^8^. The presence of such Treg-inducing neoepitopes in tumors may camouflage cancerous cells from immune surveillance. Additionally, T cells recognizing neoepitopes with "self" TCR faces may have been rendered anergic during thymic selection or deleted before they can be released to the periphery. Therefore, self-like neoepitopes may reduce overall tumor immunogenic potential.

We hypothesized that the presence of a mutation alone is not sufficient to generate an immunogenic neoepitope, but that significant differences must exist at the HLA- and/or TCR-interfaces of the neoepitope as compared to (1) the non-mutated form of the neoepitope, and (2) to other self-epitopes, in order to be recognized as non-self by the immune system. Therefore, we hypothesized that individual patient outcomes may be determined by neoepitope analyses that integrates the consideration of self-epitopes into the analysis of tumor neoantigens. To test this assumption, we analyzed large scale bladder cancer genomic data using Ancer, an automated computational immunoinformatics pipeline that we developed for neoantigen screening and vaccine design. Ancer shares components with our commercial-grade screening platforms used routinely in immunogenicity assessments of infectious disease antigens^14^, such as the EpiMatrix algorithm for HLA class I and HLA class II neoepitope identification, and the JanusMatrix algorithm for tolerated, tolerogenic, and cross-reactive T cell epitope identification^14,15^. Animal proof-of-concept studies using RNA replicons revealed that neoantigen-based cancer vaccines designed with Ancer are immunogenic, induce multifunctional CD4^+^ and CD8^+^ T cell responses, and are effective in challenge experiments^16^. The prognostic value of Ancer is demonstrated here by our analysis of genomic and clinical data derived from bladder cancer patients. Our evaluation of patient survival with Ancer shows a marked improvement over other stratification approaches such as using TMB or a quantitative assessment of neoepitopes identified with NetMHCpan 4.0 and NetMHCIIpan 3.1^17,18^, commonly used HLA class I and HLA class II T cell epitope prediction tools, respectively. Compared to existing tools, Ancer’s ability to assess self-like epitopes allows the identification of more immunologically relevant neoepitopes, which can also be employed to optimize personalized cancer vaccines.

## Results

### Neoepitope load is highly correlated with bladder cancer patient tumor mutational burden

Sequencing data from 412 chemotherapy-naïve bladder cancer (BLCA) tumors of the TCGA were downloaded and analyzed with Ancer for neoepitope identification and triaging. The TGCA's BLCA dataset was derived from a cohort of muscle-invasive bladder cancer patients who remained at large untreated prior to tumor collection^19^. HLA class I and class II types were first determined from the raw sequencing data using the xHLA, seq2HLA, and HLA-VBSeq in silico tools^20–22^. While predictions of HLA allele groups (i.e. two-digit HLA types) were largely consistent across the three HLA typing tools, some results varied when predicting specific HLA proteins (i.e. four-digit HLA types). Overall, 60% of HLA-A, 40% of HLA-B, and 67% of HLA-DRB1 protein predictions (four-digit HLA types) were concordant across the three HLA typing algorithms and a consensus approach was employed to resolve differing HLA mapping. Concordance rose to 85%, 81%, and 83% for HLA-A, HLA-B, and HLA-DRB1, respectively, when considering allele group (two-digit) results, highlighting a relatively high agreement between the HLA typing tools when predicting HLA families.

**Figure 1:**
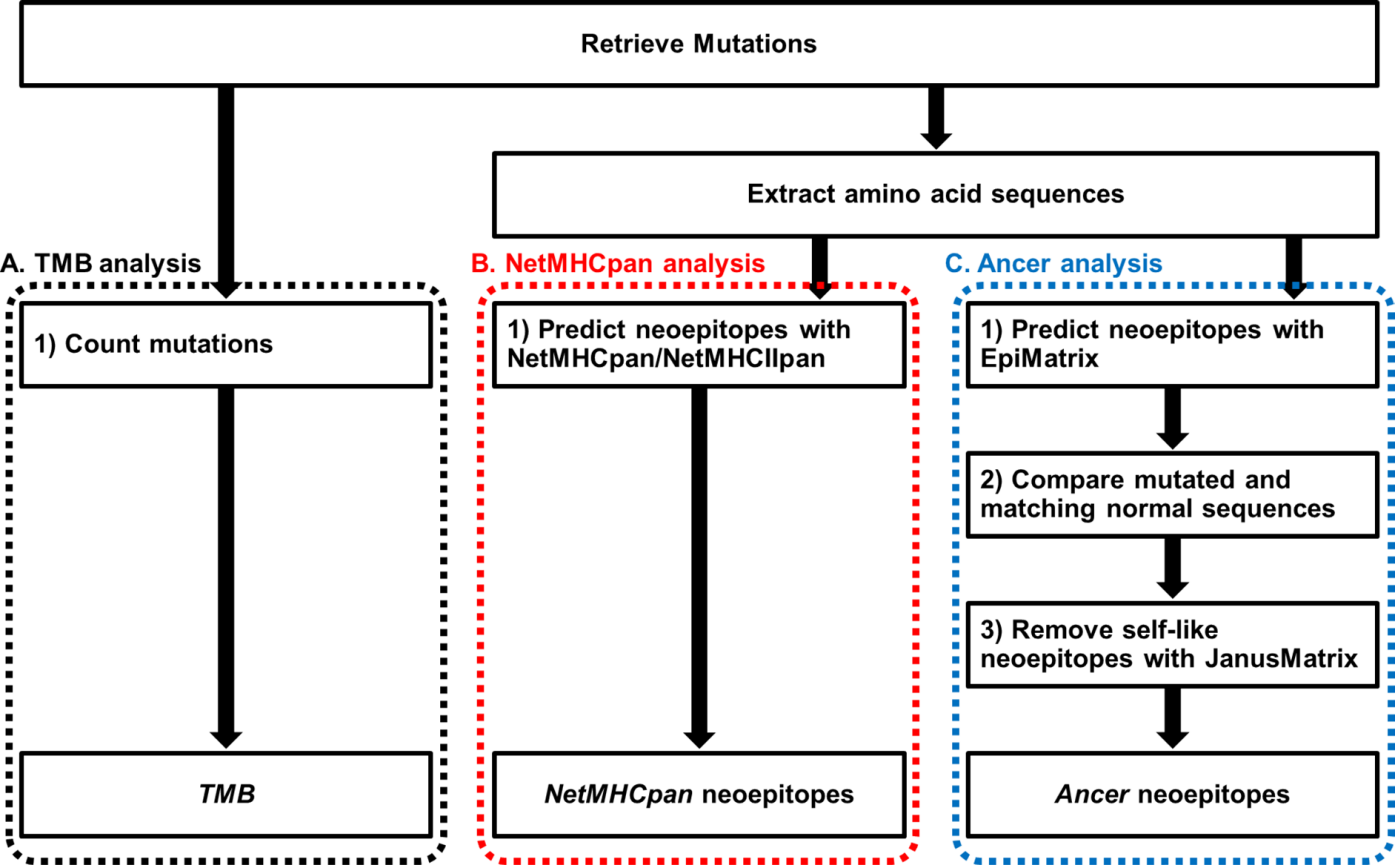
BLCA mutanome analysis workflow. Mutations were retrieved for each patient sample and evaluated using three analysis workflows and then compared for overall survival and disease-free survival predictive accuracy. The three types of analyses were defined as follows: (**A**) “TMB analysis”: tumor mutational burden is evaluated from the count of mutations present in each tumor. (**B**) “NetMHCpan analysis”: mutation-bearing HLA class I and HLA class II ligands are identified with NetMHCpan 4.0 and NetMHCIIpan 3.1, respectively. This approach is similar to the one employed by the TCGA Research Network in their analysis of the BLCA cohort^19^. (**C**) “Ancer analysis”: mutation-bearing HLA class I and HLA class II ligands are identified with EpiMatrix, compared to matched normal sequences to identify Ancer-defined neoepitopes, and filtered with JanusMatrix to remove neoepitopes homologous to self.

Cancer mutanomes were subsequently analyzed with the Ancer pipeline to evaluate HLA class I and HLA class II neoepitope burdens. Key steps in Ancer includes (1) identification of HLA class I and HLA class II mutation-bearing epitopes, or neoepitopes, with the EpiMatrix algorithm, (2) comparison of mutated and matched normal sequences for HLA/TCR-faces comparison to refine neoepitopes and discard ones where mutations do not significantly alter normal sequences, and (3) in-depth homology analysis of neoepitope TCR-faces against other self-antigens using the JanusMatrix algorithm to remove self-like cross-reactive, tolerated, or actively tolerogenic neoepitopes (Fig. 1). In this last step, each predicted HLA ligand is analyzed two ways: by evaluating its constitutive agretope (or HLA-facing interface) as well as its epitope (or TCR-facing interface). Ligands derived from the human proteome that have the identical TCR face and a similar-binding (but not necessarily sequence-identical) agretope are returned. We expect that T cells interacting with commonly observed TCR faces are deleted during thymic selection or are developed into cells that have a regulatory phenotype. Hence, epitopes presenting these commonly occurring TCR faces, or self-like epitopes, may be tolerated or actively tolerogenic.

**Figure 2:**
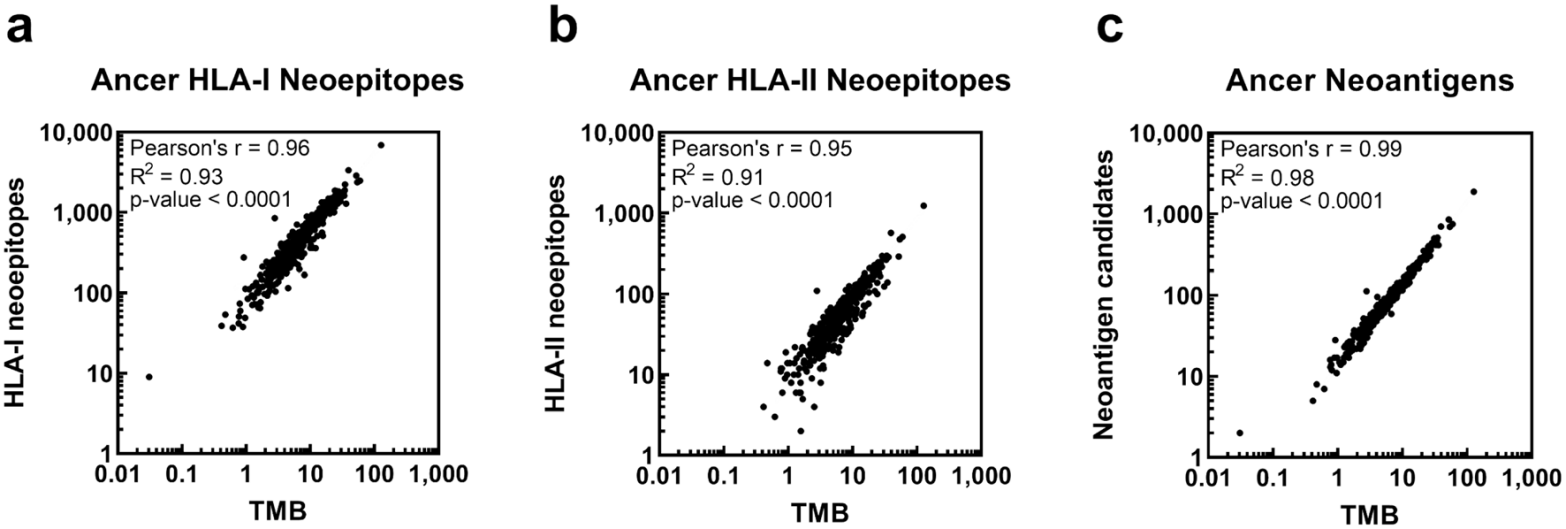
Association between mutational and neoepitope landscapes. BLCA mutanomes were analyzed with Ancer to determine counts of Ancer-defined HLA class I neoepitopes (**a**), Ancer-defined HLA class II neoepitopes (**b**), and Ancer-defined neoantigen candidates that could be used for precision immunotherapy purposes (**c**). Numbers of HLA class I neoepitopes, HLA class II neoepitopes, and neoantigen candidates observed in each patient is strongly correlated with observed tumor mutational burden (TMB). Each dot corresponds to one TGCA BLCA patient.

We next aimed to identify the distribution of Ancer-derived neoepitopes across TCGA BLCA patients. HLA class I and class II Ancer neoepitopes were identified in all but one and three BLCA patients, respectively (Table 1, Supplementary Data 1). The median number of Ancer HLA class I neoepitopes was 400, and the median number of Ancer HLA class II neoepitopes was 54. As expected, patient total TMB was strongly correlated with the total counts of Ancer HLA class I (Pearson's r = 0.96, *p* < 0.0001) and class II neoepitopes (Pearson's r = 0.95, *p* < 0.0001) (Fig. 2a, b).

**Table 1:** Characteristics of the TCGA BLCA cohort.

Characteristic	TCGA BLCA Cohort (N = 412) median [range]
Age-years	69 [34–90]
Female sex-count (%)	108 (26%)
DFS-months	30.1 [0.4–163.2]
OS-months	34.0 [0.4–165.9]
TMB-count per Mb	
Silent	1.6 [0–26.3]
Non-silent	4.7 [0–101.7]
Total	6.5 [0–128.0]
PD-L1 expression-FPKM	0.93 [0.05–35.16]
Mutation analyzed-counts	145.5 [0–3060]
Ancer HLA class I neoepitopes-counts	400 [0–6892]
Ancer HLA class II neoepitopes-counts	54 [0–1232]
Candidate neoantigens (Ancer-based)-counts	90 [0–1888]

We then estimated the landscape of neoantigens that would be suitable for a hypothetical vaccine formulation using the Ancer tool. Candidate vaccine antigens were defined by Ancer based on a series of automated instructions that created optimal amino acid sequences, usually ranging between 15 and 25 amino acids, that contained overlapping HLA class I and class II neoepitopes of interest, while avoiding the inclusion of cross-conserved or otherwise detrimental epitopes, along with flanking residues. We again found that the number of Ancer-designed neoantigen candidates was strongly correlated with patient total TMB (Pearson's r = 0.99, *p* < 0.0001) (Fig. 2c). As most neoantigen-based vaccine trials employ up to 20 neoantigen candidates^23,24^, we determined that at least 20 optimal sequences could be generated for patients that have 1.46 mutations per megabase or more, corresponding to 95% of the BLCA cohort. Therefore, most bladder cancer patients present a sufficiently high number of mutations and would be eligible for standard neoantigen-based vaccinations designed by Ancer.

### Number of Ancer-derived neoepitopes is a prognostic biomarker for bladder cancer

We next evaluated whether neoepitope count was a prognostic biomarker in bladder cancer and compared the performance of the Ancer pipeline analysis with TMB or neoepitope counts determined with NetMHCpan 4.0 and NetMHCIIpan 3.1. As no standardized NetMHCpan-based neoantigen computational pipeline exists, we employed an approach that was similar to the one employed by the TCGA Research Network in their analysis of the BLCA cohort^19^, where neoepitopes were defined as mutated HLA ligands identified with default NetMHC- pan cutoff values. Our latter analysis, employing NetMHCpan 4.0 and NetMHCIIpan 3.1, is referred to as the "NetMHCpan" analysis in this manuscript (Fig. 1).

The cohort's median TMB was employed to identify BLCA patients with high (TMB^hi^) or low TMB (TMB^lo^). Similarly, patients with high and low neoepitope burdens were defined using the median class I or class II neoepitope counts, based on the Ancer analysis or the NetMHCpan analysis (Fig. 1). Patients with overall high neoepitope burdens were defined as having both a higher than median class I neoepitope burden and a higher than median class II neoepitope burden (CD8^hi^CD4^hi^ patients). These patients' survival was compared to the remainder of the cohort, which includes (1) patients with high class I neoepitope burden but lower than median class II neoepitope burden (CD8^hi^CD4^lo^ patients), and (2) patients with lower than median class I neoepitope burden, regardless of their class II neoepitope burden (CD8^lo^ patients, i.e. CD8^lo^CD4^lo^ and CD8^lo^CD4^hi^ patients). The use of categorical variables was motivated by the desire to generate distinct patient subgroups combining CD8 and CD4 neoepitope information without losing information about the source of the neoepitopes (class I vs class II). Adding counts of CD8 and CD4 neoepitopes to generate an "overall" neoepitope burden would obscure information that we believe is important to consider and would collapse epitopes that are associated with different immunological functions, such as promoting either cytotoxic (class I) or helper (class II) T cell responses.

**Figure 3:**
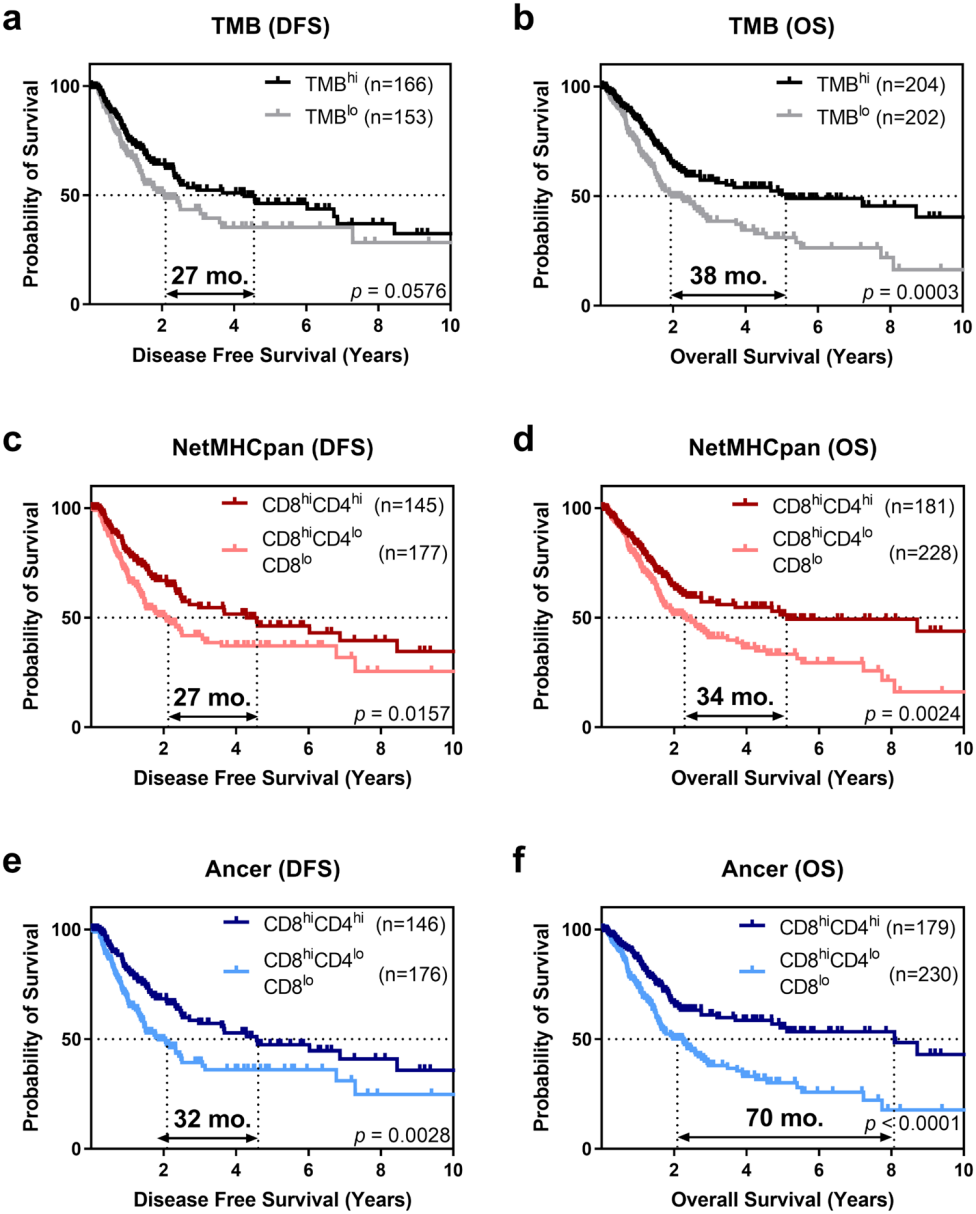
Stratification of cancer patients according to TMB analysis, NetMHCpan analysis, and the Ancer pipeline. TCGA bladder cancer patients were separated based on their median TMB (**a**,**b**), NetMHCpan neoepitope burden (**c**,**d**), or Ancer pipeline-defined neoepitope burden (**e**,**f**). Median disease-free survival (DFS) (**a**,**c**,**e**) and median overall survival (OS) (**b**,**d**,**f**) were evaluated with the Kaplan–Meier estimator. Double-ended arrows define the differences in median survival times between the groups for each of the analyses. Statistical significance was evaluated using the log-rank test. The largest differential in median overall survival, 70 months, was obtained with the Ancer pipeline and was more than double the difference in median overall survival observed when stratifying patients using NetMHCpan (34 months).

**Figure 4:**
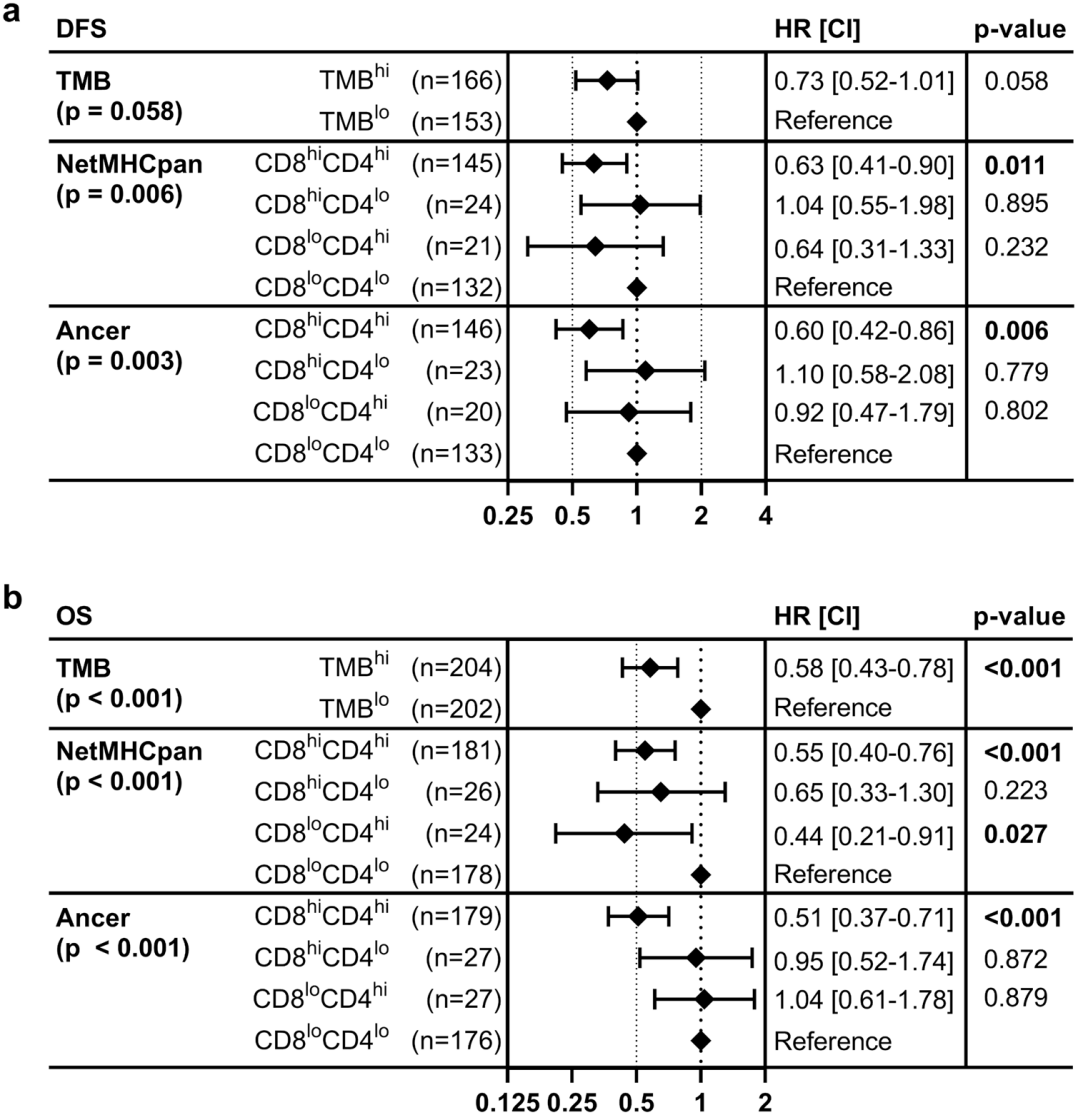
Univariate survival analysis forest plots. Univariate survival analyses were conducted separately for the TMB, NetMHCpan, and Ancer analyses while considering either disease free survival (DFS) (**a**) or overall survival (OS) (**b**). BLCA patients were separated based on their median TMB or neoepitope burdens. Association with DFS and OS is improved with Ancer compared to the other analysis. Hazard ratios (HR), confidence intervals (CI) and *p*-values (*p*) were calculated using univariate Cox proportional-hazard models. Overall log-rank *p*-value is provided for each model on the left.

While the difference in DFS between TMB^lo^ and TMB^hi^ patients was not significant (Fig. 3a), CD8^hi^CD4^hi^ patients, defined by Ancer or NetMHCpan, had a significantly prolonged DFS (Fig. 3c, e). The maximum difference in median DFS was achieved by defining neoepitopes and removing tolerated or tolerizing neoepitopes with the Ancer pipeline. Ancer-derived neoepitope quantification resulted in a DFS difference of 32 months (log-rank *p* = 0.0028), compared with 27 months when using NetMHCpan-derived neoepitopes (log-rank *p* = 0.0157). Improved patient stratification with Ancer was also confirmed using Cox proportional-hazards models when considering the CD8^hi^CD4^hi^, CD8^hi^CD4^lo^, CD8^lo^CD4^hi^, CD8^lo^CD4^lo^ patient subgroups (Fig. 4a).

Univariate analyses focusing on overall survival showed that TMB^hi^ patients or CD8^hi^CD4^hi^ patients, based on the NetMHCpan or the Ancer analyses, had statistically prolonged survival compared to the remainder of their respective cohorts (Fig. 3b, d, f). However, improved patient cohort differentiation was again achieved using the Ancer pipeline (log-rank *p* < 0.0001), when compared to stratifications performed using median TMB (log-rank *p* = 0.0003) or median NetMHCpan neoepitope burden (log-rank *p* = 0.0024). The largest differential in median overall survival was obtained with the Ancer pipeline and was more than double the difference in median overall survival observed when stratifying patients using NetMHCpan (70 versus 34 months). Cox proportional-hazards models for OS confirmed improved hazard ratios for the CD8^hi^CD4^hi^ patients with the stratification performed with Ancer compared to the other analyses (Fig. 4b).

### HLA- and TCR-face assessments using the Ancer pipeline improves neoepitope quality

The unique method of neoepitope characterization is one of the key differentiating features of the mutanome analysis that is performed using the Ancer pipeline. First, for each predicted neoepitope, Ancer performs a comparison of its HLA- and TCR-facing portions against its respective normal sequence to evaluate the impact of the underlying mutation on either faces (Ancer pipeline step 2, Fig. 1). Once the unique neoepitope is confirmed to be truly "neo" (i.e. not matching to the normal sequence), the JanusMatrix algorithm filters out any neoepitope that shares a high degree of homology, at the TCR interface, with other self-epitopes (Ancer pipeline step 3, Fig. 1). These two filters have the effect of removing from consideration neoepitopes that may not contribute to productive anti-tumor immune responses.

To test the effect of these filters, we first determined the number of "raw" Ancer class I and class II neoepitopes contained within mutated sequences of bladder cancer patients, i.e. without comparing predicted neoepitopes to their matched normal sequence or other self-antigens (i.e. step 1 only from the Ancer pipeline outlined in Fig. 1 and skipping steps 2 and 3). Then, we determined the number of "non-matching" Ancer class I and class II neoepitopes that significantly differed from their matched normal sequences, but without filtering them using the JanusMatrix algorithm (i.e. steps 1 and 2 from the Ancer pipeline detailed in Fig. 1 and skipping step 3). Finally, stratification of bladder cancer patients was performed based on (1) median "raw" Ancer neoepitope burdens (step 1 of the Ancer pipeline), (2) median "non-matching" Ancer neoepitope burdens (steps 1 and 2 of the Ancer pipeline), and (3) median Ancer neoepitope burdens (all steps of the Ancer pipeline).

**Figure 5:**
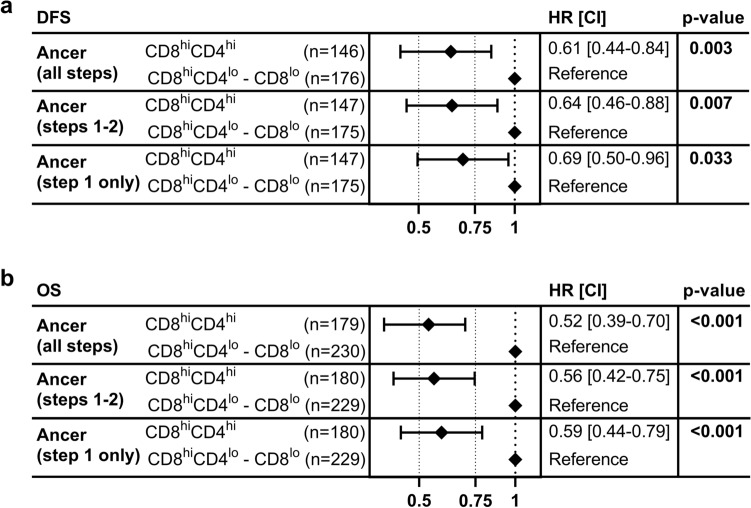
Effect of Ancer's neoepitope and homology filters on survival analyses. Ancer neoepitope and homology filters improve association with disease free survival (DFS) (**a**) and overall survival (OS) (**b**). Hazard ratios (HR), confidence intervals (CI) and *p*-values (*p*) were calculated using univariate Cox proportional-hazard models. BCLA patients were separated based on their Ancer neoepitope burden (Ancer, all steps), similarly to Fig. 3, "non-matching" Ancer neoepitope burden without considering the JanusMatrix filter (Ancer, steps 1–2), or based on their "raw" Ancer neoepitope burden (Ancer, step 1 only).

Significant associations with DFS (Fig. 5a) and OS (Fig. 5b) were observed when stratifying bladder cancers based on their raw Ancer class I and class II neoepitope burdens (Ancer step 1 only; DFS HR = 0.69, *p* = 0.033 OS HR = 0.59, *p* < 0.001). Gradual improvements were obtained in subsequent steps of the analysis pathway, which considered comparisons with matched normal sequences (Ancer steps 1–2; DFS HR = 0.64, *p* = 0.007; OS HR = 0.56, *p* < 0.001), and other self-antigens (Ancer steps 1–3; DFS HR = 0.61, *p* = 0.003; OS HR = 0.52, *p* < 0.001). The incremental enhancement in hazard ratios suggests that the quality of neoepitopes retained after each filtering step is improved by removing sequences that do not contribute to the tumor's immunogenicity. Consequently, using all steps of the pipeline best predicted patients’ survival.

### Improved neoepitope quality is associated with enhanced five-year survival prediction of bladder cancer patients

To evaluate Ancer's ability to identify long-term survivors based on their genomic data, we hypothesized that bladder cancer patients with high Ancer class I and class II neoepitope burdens (Ancer CD8^hi^CD4^hi^ patients) would be more likely to survive more than five years while other patients would survive less than five years. Predicted survival status was compared to observed overall survival for 220 BLCA patients, after removing 192 individuals lost to follow-up before the five-year mark and for which survival status could not be precisely assessed.

**Figure 6:**
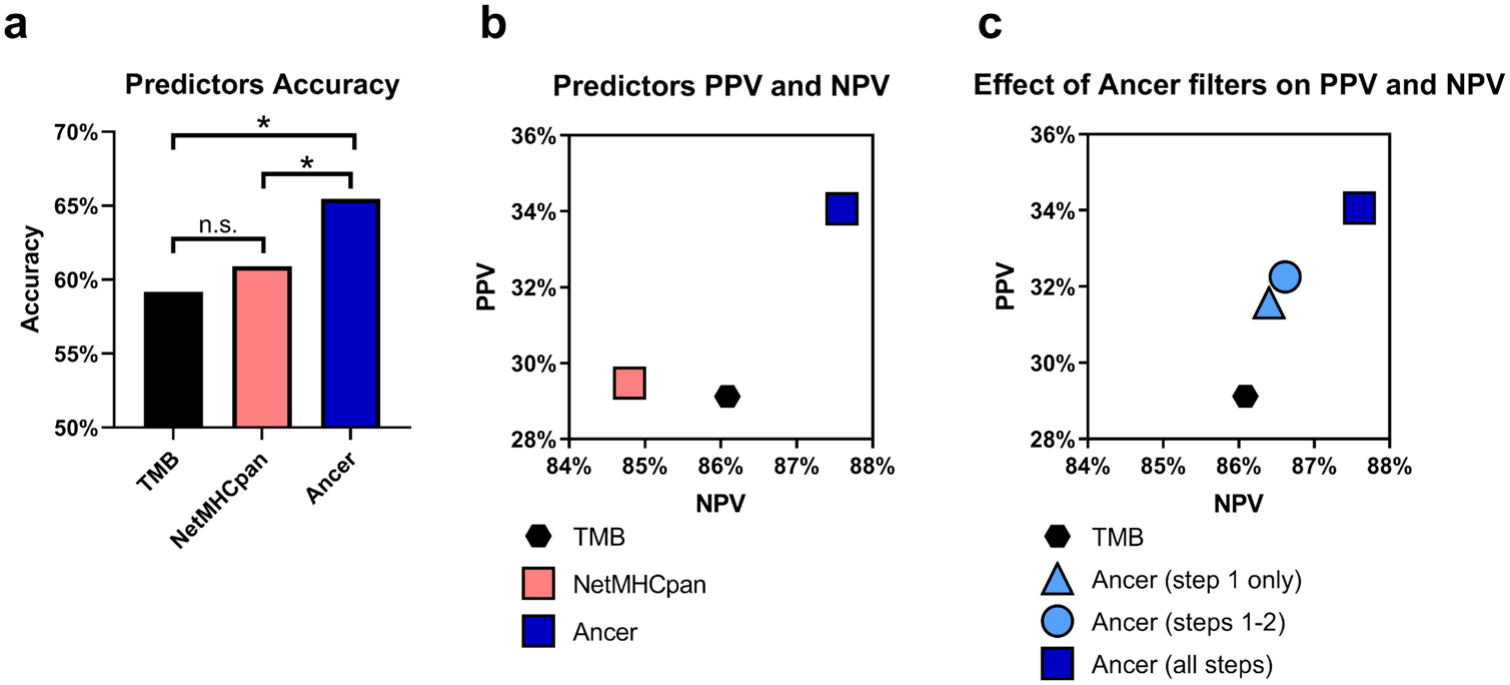
Prediction of bladder cancer patient five-year survival rate. Ancer pipeline analysis improves prediction of five-year survival compared to TMB- or NetMHCpan-based predictors. BLCA patients were predicted to survive more or less than five years based on their mutational or neoepitope burdens. Predicted survival status was compared to observed overall survival. (**a**) Accuracy of the five-year survival predictions. *p-value < 0.05, McNemar's test. (**b**) Positive (PPV) and negative predictive values (NPV) obtained for the TMB, NetMHCpan, and Ancer predictors. (**c**) PPVs and NPVs obtained using truncated and full versions of the Ancer pipeline. The increased NPV as compared to NetMHCpan and TMB suggests that an analysis that identifies tolerated or tolerogenic epitopes (Ancer) may be better suited to identify patients at a greater risk of earlier mortality (~ 9 out of 10 correct predictions) than the other types of analyses.

For the cohort of 220 bladder cancer patients with known five-year OS outcomes, Ancer neoepitope burden, as determined by the full Ancer pipeline, was a more accurate predictor of five-year survival (Fig. 6a, 65% accuracy) than TMB (59% accuracy) or NetMHCpan neoepitope burden (61% accuracy). The Ancer analysis also achieved higher Positive Predictive Value (PPV) and Negative Predictive Value (NPV) statistics (PPV = 34%, NPV = 88%) as compared to TMB- (PPV = 29%, NPV = 86%) or NetMHCpan-based predictors (PPV = 29%, NPV = 85%) (Fig. 6b). The elevated NPV obtained with Ancer suggests that our analysis may be better suited to identify patients at a greater risk of earlier mortality (~ 9 out of 10 correct predictions).

By isolating each of the Ancer pipeline steps in this five-year survival analysis, we confirmed the additive importance of Ancer's unique homology filters (steps 2 and 3 from Fig. 1) which demonstrated gradual improvements in PPV and NPV upon their integration into the five-year survival predictor (Fig. 6c). This result further showcases the improvement in predictive capacity that results from refinement of neoepitopes by using JanusMatrix to eliminate putatively tolerated or tolerogenic neoepitopes that may not contribute to tumor immunogenicity. Since Ancer employs more than one variable (i.e. both HLA class I and class II neoepitope burden), generating Receiver Operating Characteristic (ROC) curves was not possible. Instead we replicated our five-year survival analysis for various survival intervals, every 3 months between t = 0 and t = 14 years, and calculated PPVs and NPVs for each analysis and time interval. The resulting values were plotted to calculate AUCs for each analysis (Supplementary Fig. 1). The AUC obtained for Ancer (AUC = 0.6506) was greater than those obtained for the TMB (AUC = 0.6270) and NetMHCpan (AUC = 0.5991) analyses, again demonstrating superior classification using Ancer across a range of survival periods.

### Multivariate analysis indicates Ancer neoepitope burden is independently predictive of DFS and OS

The robustness of Ancer as a prognostic biomarker was evaluated in multivariate analyses to test if the association between Ancer CD8 and CD4 neoepitope burdens and patient outcome remained significant after adjusting for common cofactors, such as TMB, age, sex, PD-L1 expression, smoking status, and disease stage. A comparative analysis was performed with NetMHCpan CD8 and CD4 neoepitope burdens.

We first evaluated each clinical cofactor in separate univariate analyses to identify which of them were significantly associated with patient survival. Cofactors significantly associated with survival in univariate analyses were subsequently included in multivariate analyses. Of those, only disease stage was significantly associated with DFS (Table 2, *p* < 0.001). With respect to OS, age (*p* < 0.001) and disease stage (*p* < 0.001) were significant cofactors. The significance of the TMB, NetMHCpan, and Ancer factors in these univariate analyses were maintained whether considered as categorical or continuous variables. Other factors taken into consideration (sex, PD-L1 expression, smoking status) did not reach statistical significance in any of the univariate analyses.

**Table 2:** Exploration of other factors associated with survival.

Endpoint	Factor	Units/categories	Log-rank *p*-value
DFS	TMB	TMB^hi^ TMB^lo^	0.058
	**NetMHCpan**	CD8^hi^CD4^hi^ CD8^hi^CD4^lo^ and CD8^lo^	**0.016**
	**Ancer**	CD8^hi^CD4^hi^ CD8^hi^CD4^lo^ and CD8^lo^	**0.003**
	Age	Years	0.083
	Sex	Female Male	0.589
	PD-L1 expression	FPKM	0.137
	**Disease stage**	Stage I and II Stage III Stage IV	**< 0.001**
	Smoking status	Current smoker Former smoker Never smoker	0.163
OS	**TMB**	TMB^hi^ TMB^lo^	**< 0.001**
	**NetMHCpan**	CD8^hi^CD4^hi^ CD8^hi^CD4^lo^ and CD8^lo^	**0.002**
	**Ancer**	CD8^hi^CD4^hi^ CD8^hi^CD4^lo^ and CD8^lo^	**< 0.001**
	**Age**	Years	**< 0.001**
	Sex	Female Male	0.492
	PD-L1 expression	FPKM	0.073
	**Disease stage**	Stage I and II Stage III Stage IV	** < 0.001**
	Smoking status	Current smoker Former smoker Never smoker	0.130

Each factor was analyzed in a univariate survival analysis to determine its significance with disease free (DFS) or overall survival (OS). Significant factors and log-rank p-values are bolded.

**Figure 7:**
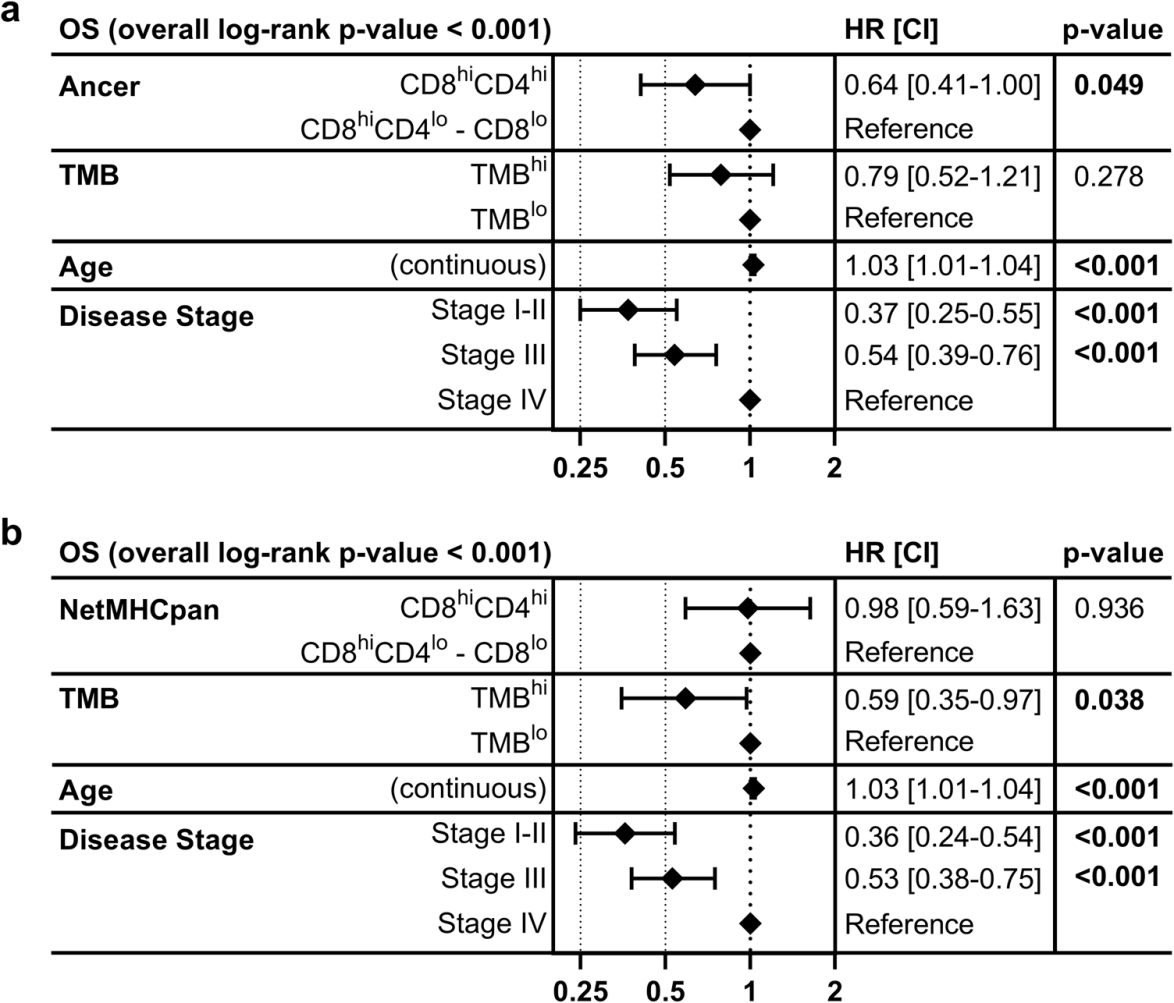
Multivariate survival analysis forest plots. Ancer neoepitope burden remains a significant co-factor associated with overall survival when adjusting for TMB, age, and disease stage (**a**). NetMHCpan neoepitope burden's association with overall survival is lost when adjusting for TMB, age, and disease stage (**b**). Hazard ratios (HR), confidence intervals (CI) and *p*-values (*p*) were calculated using multivariate Cox proportional-hazard models. Ancer neoepitope burden remained a significant cofactor associated with overall survival once adjusted for TMB, age, and disease stage.

Multivariate survival models were subsequently generated to investigate whether Ancer or NetMHCpan neoepitope burdens remained associated with DFS when adjusting for TMB and disease stage, or associated with OS when adjusting for TMB, age, and disease stage. We expected to lose association with survival as we had previously observed strong correlations between TMB and counts of HLA class I and class II neoepitopes (Fig. 2). Ancer and NetMHCpan neoepitope burdens' association with DFS was lost when adjusting for TMB and disease stage (data not shown). Nonetheless, Ancer neoepitope burden remained a significant cofactor associated with OS once adjusted for TMB, age, and disease stage (Fig. 7a). Independence was maintained in analyses where Ancer neoepitope burden was considered as a continuous variable. TMB was no longer significantly associated with OS in this model. Multivariate survival analyses including NetMHCpan neoepitope burden no longer showed significant association with OS after a similar adjustment (Fig. 7b).

## Discussion

In this study, we report the application of Ancer, a novel multistep immunoinformatic pipeline for the identification and refinement of neoepitopes most likely to generate an effector T cell immune response. We demonstrate that Ancer improved prediction of clinical outcomes in bladder cancer compared with existing neoepitope identification tools. While these findings have broader relevance, we focused our analyses in this study on bladder cancer given that this disease has long been known to elicit an endogenous anti-tumor response with known individual tumor variability at the patient level. Furthermore, this is a cancer of growing worldwide importance causing serious morbidity and mortality worldwide.

In the landmark study of the TCGA bladder cancer (BLCA) cohort, Robertson et al*.* showed that TMB and HLA I neoepitopes burden, identified with NetMHCpan 3.0, were associated with BLCA patient survival^19^, even though these patients did not undergo checkpoint inhibition immunotherapy as this form of therapy was not approved in bladder cancer at the time the samples were collected. In addition to quantity, other groups have focused on the quality of predicted neoepitopes, highlighting a link between patient outcome and the presence of high quality neoepitopes, ones homologous to other known immunogenic epitopes derived from infectious agents^5,6^. While many T cell epitope induces immunogenic effector T cell responses, some may instead engage regulatory T cells (Tregs) leading to tolerance and immunosuppression^25–27^. Treg epitopes, or Tregitopes, have been documented in both biologics and pathogens^10,11,28,29^. Mutations generating Treg neoepitopes, or neo-Tregitopes, may camouflage tumors from the immune system. Hence, they should be filtered or removed when optimally evaluating tumor T cell neoepitope burdens or when designing novel neoantigen-based precision immunotherapies.

Ancer integrates EpiMatrix, an extensively validated HLA class I and HLA class II T cell epitope prediction algorithm, in addition to JanusMatrix, a specialized homology tool to identify putative tolerated, cross-reactive, or tolerogenic (i.e. Treg) epitopes (Fig. 1). These tools have been well validated in the biologics and infectious disease fields^10–12,14,29–31^, and have been employed in tumor associated antigen and neoantigen-based vaccine studies^16,32^. Analysis of genomic data from the TCGA BLCA cohort with Ancer found that both Ancer HLA class I and HLA class II neoepitope loads were strongly correlated with patient TMB (Fig. 2a, b), similarly to other reports using alternative T cell epitope prediction tools^19^. Our analysis also suggests that Ancer can be used as a feasible adjunct for developing personalized vaccines for bladder cancer patients (Fig. 2c), despite the relatively lower number of mutations compared to tumors traditionally investigated for neoantigen-based therapy, such as melanoma^23,33^.

When we stratified BLCA patients based on their HLA I and HLA II neoepitope burden, we observed significantly prolonged disease free and overall survival in patients whose tumor contains both high numbers of HLA I and HLA II neoepitopes (CD8^hi^CD4^hi^ patients), compared to other individuals (Fig. 3e, f). Stratifications performed with Ancer were superior to comparative analyses performed with TMB or with neoepitopes counts determined by commonly used T cell epitope prediction tools (Figs. 3, 4). In addition, we showed that Ancer's precise epitope filtering and characterization steps contributed to this increased association with survival, by removing from consideration neoepitopes that should not support T cell-based recognition of the tumor based on homology with matched normal and other self-sequences (Fig. 5). Refining tumor neoepitope burdens by discarding putative non-immunogenic or putative inhibitory T cell neoepitopes provided a clear advantage at improving our understanding of patient outcomes. Follow-up analyses investigating how the balance between putative effector and regulatory neoepitopes affected survival did not yield conclusive results (data not shown).

These observations led us to test the assumption that long-term bladder cancer survivors could be identified by evaluating their tumor for immunogenic neoepitope content. Five-year survival classification of BLCA patients based on Ancer HLA I and HLA II neoepitope contents appeared again to be superior compared to classifications based on NetMHCpan neoepitope content or based on TMB (Fig. 6a, b, Supplementary Fig. 1). Furthermore, we showed that each filtering steps embedded in Ancer incrementally refined neoepitopes quality which subsequently improved five-year survival assessments (Fig. 6c). Lastly, our analysis suggests Ancer neoepitope content remained a significant factor in patient overall survival even when adjusted for TMB, and other clinical covariates such as age at diagnosis and disease stage (Fig. 7). It was initially unexpected that Ancer remained significant when adjusting for TMB, given the high correlation observed between TMB and counts of class I neoepitopes (Fig. 2a) and class II neoepitopes (Fig. 2b). However, Ancer employs a combination of both counts of class I and class II neoepitopes, which increases the precision of the classifier over TMB. Upon close inspection of the results, 54 BLCA TCGA patients, or 13% of the cohort, are classified differently by the TMB and Ancer analyses, with 14 TMB^lo^ patients classified as CD8^hi^CD4^hi^ patients by Ancer, and 40 TMB^hi^ patients classified as CD8^hi^CD4^lo^/CD8^lo^ patients by Ancer. These observations further support the concept of evaluating both class I and class II neoepitope content in prognostic analyses.

There are some limitations to our data. While bladder cancer patients with "high" and "low" neoepitope burdens were identified according to median number of neoepitopes identified in the TCGA BLCA cohort, alternative cutoffs may be more appropriate to further identify specific patients that are at an even higher risk of disease recurrence or death based on their mutanome, and for whom more aggressive treatment options may be considered. Nonetheless, a similar improvement with Ancer over traditional methods was observed when using continuous variables. Furthermore, our current analysis focuses on patients who did not undergo checkpoint inhibitor (CPI) therapy and follow-up analyses are ongoing to determine whether the multi-step filtering process used in the Ancer pipeline will also predict for CPI-treated patients. We hypothesize that filtering for ‘true’ neoepitopes and removing tolerated neoepitopes may also be critical for understanding response to checkpoint therapy and for determining predicted outcomes of patients treated with a CPI agent.

In summary, our report suggests that optimal host-immune recognition of CD8, CD4, and Treg neoepitopes plays a key role in endogenous cancer control and duration of survival. These results suggest that defining the number of true neoepitopes using Ancer may represent a novel prognostic or predictive biomarker. In addition to biomarker identification, using Ancer when designing novel precision immunotherapies, such as neoantigen-based vaccines or TCR-based therapies, offers the advantage of prioritizing immunogenic CD8 and CD4 neoepitopes, while discarding self-like or inhibitory neoepitopes. Therapies that include these design considerations should promote an optimal immune response in cancer patients, leading to improved clinical outcomes when combined with checkpoint inhibitors. The advantage of Ancer-designed precision immunotherapies will be determined in forthcoming clinical trials.

## Methods

### HLA typing for TCGA samples

In order to perform HLA typing, the full set of normal sequencing data was obtained from The Cancer Genome Atlas (TCGA) for its bladder cancer (BLCA) cohort including blood-derived normal samples and solid tissue normal samples, for each patient. HLA genes all occur in a continuous, approximately 5 megabase region of chromosome 6, and for efficiency only this segment of the aligned read files was retrieved from the genomic data source. Performance using entire BAM file versus the segment was validated and found to be similar. Biobambam2 (version 2.0.89) was used to convert aligned reads to paired read FASTQ files. Bwa (version 0.7.17) was used to align reads to HLA allele references as input for HLA-VBSeq. We first used xHLA and seq2HLA. These two tools represented alternative methodologies for calling HLA types. xHLA called the three class I types (HLA-A, HLA-B, and HLA-C) and 3 of the class II genes (HLA-DPB1, HLA-DQB1, HLA-DRB1), while seq2HLA called three class I genes, 6 class II genes, and 9 non-classical class II genes. The majority of calls were in agreement for class I and class II, except for HLA-DPB1. We then used HLA-VBSeq to form a consensus classification of HLA class I and II types.

### Tumor mutational burden (TMB) analysis

Counts of silent and non-silent mutations per megabase for the TCGA bladder cancer cohort were retrieved from NCI's Genomic Data Commons^34^. Patients whose combined silent and non-silent TMB fell above or below the cohort median were defined as having high (TMB^hi^) or low (TMB^lo^) TMB, respectively.

### Neoepitope analysis

Somatic mutations were retrieved from the TCGA for the bladder cancer cohort. Mutations identified through all available variant callers (Muse, Mutect, SomaticSniper, and VarScan) were first merged for each patient. Mutations were subsequently analyzed with two independent analyses: (1) with an internally designed neoantigen pipeline, Ancer, that uses proprietary T cell epitope prediction tools and (2) with publicly available T cell epitope prediction tools, NetMHCpan 4.0 and NetMHCIIpan 3.1^17,18^.

Ancer, an end-to-end computational platform that analyzes mutanome data, identifies patient-specific T cell neoepitopes, and subsequently rank them for immunotherapy design. Ancer neoantigen analyses can be performed through collaborations with EpiVax Therapeutics, Inc. Readers are encouraged to contact the authors if they wish to use Ancer in their research.

Ancer makes use of the EpiMatrix and JanusMatrix algorithms for T cell epitope mapping and removal of putative inhibitory or cross-reactive epitopes, respectively. Both tools have been previously described for the immunogenicity analysis of biologics (ISPRI) and other non-mutated vaccine antigens (iVAX)^14^. Briefly, Ancer parses mutated and matched normal amino acid sequences into overlapping 9- and 10-mer frames. Each frame is then assessed with EpiMatrix to determine its likelihood of binding to one of a patient HLA class I (HLA-A, HLA-B) or class II (HLA-DRB1) alleles. Mutated and normal matched sequences are then compared to identify tumor-specific neoepitopes that significantly differ from their normal matched counterparts at the HLA- and/or TCR-interfaces. Neoepitopes are then screened with JanusMatrix to remove sequences cross-conserved at the TCR interface with epitopes present in self, non-mutated, proteins which may be recognized by natural regulatory T cells (Tregs) or otherwise tolerated due to negative selection of lymphocytes recognizing self-antigens. JanusMatrix has previously been employed to identify Treg epitopes in HCV and H7N9 influenza^10–12^ among other targets. For immunotherapy design, EpiMatrix and JanusMatrix results are compiled for each patient and then reviewed by Ancer to computationally design neoantigen sequences that only contain neoepitopes with limited potential to cross-react with self-epitope sequences.

HLA Class I and Class II neoepitope counts were calculated for each patient. For the Ancer analysis, counts were obtained before and after filtering neoepitopes with the JanusMatrix algorithm, which removes putative tolerated, tolerogenic, or cross-conserved epitopes.

For the NetMHCpan analysis, neoepitopes were defined as mutated epitopes predicted to bind to patients' HLA according to recommended thresholds (Class I/NetMHCpan 4.0: below a percentile rank of 2; Class II/NetMHCIIpan 3.1: below a predicted binding affinity of 500 nM), similarly to the methodology employed by the TCGA Research Network in their analysis of the same cohort of patients^19^. As neoepitopes analyses are restricted to the HLA-A, HLA-B, and HLA-DBR1 genes within the Ancer pipeline, we applied the same restrictions when analyzing mutations with NetMHCpan and NetMHCIIpan for comparative purposes.

Median neoepitope counts were employed to define patients with high and low neoepitope burdens, similarly to the TMB analysis. Patients whose count of Class I neoepitopes fell (1) at or above or (2) below the cohort median were defined as having high (CD8^hi^) or low (CD8^lo^) Class I burden, respectively. Patients whose count of Class II neoepitopes fell above or below the cohort median were defined as having high (CD4^hi^) or low (CD4^lo^) Class II burden, respectively.

### PD-L1 expression

RNA sequencing data for the TCGA bladder cancer cohort was downloaded from the TCGA. PD-L1 expression was obtained by retrieving FPKM (Fragments Per Kilobase Million) values for the ENSG00000120217.12 Ensembl Gene ID.

### Survival analysis

Clinical data, including disease free survival (DFS) and overall survival (OS), for the TCGA bladder cancer cohort was retrieved from the TCGA. Survival curves were plotted using the Kaplan–Meier estimator for the TMB (TMB^hi^ vs TMB^lo^ patients), NetMHCpan (CD8^hi^CD4^hi^ vs CD8^hi^CD4^lo^/CD8^lo^ patients), and Ancer (CD8^hi^CD4^hi^ vs CD8^hi^CD4^lo^/CD8^lo^ patients) analyses. Differences in median overall survival were evaluated with the Log-rank test. Cox proportional hazards models were employed to obtain hazard ratios for each subgroup. Clinical covariates (age, sex, PD-L1 expression, smoking status, and disease stage) were individually evaluated with Cox proportional hazards models to identify variables significantly associated with either DFS or OS. Significant cofactors were included in multivariate survival analyses that considered TMB and Ancer neoepitope groupings, or TMB and NetMHCpan neoepitope groupings. All statistical analyses were performed with GraphPad Prism and R.

## Supplementary Information

Supplementary Information 1.

Supplementary Information 2.

## Data Availability

Data used in this study, including clinical outcomes, tumor mutational burdens, and neoepitope counts, are provided in the Supplementary Source Data.
