# Supplementary material for: Multi-step screening of neoantigens’ HLA- and TCR-interfaces improves prediction of survival
Source: Sci Rep. 2021 May 11;11:9983. doi: 10.1038/s41598-021-89016-7 (PMC8113358; doi:10.1038/s41598-021-89016-7)
Supplement: Supplementary file 2 — Supplementary Information 2. [file 41598_2021_89016_MOESM2_ESM.docx]

**Supplemental Data 1. Survival Analysis Dataset.**

Data employed to perform all survival analyses, including clinical (sex, age, disease stage, smoking status, disease free survival (DFS), DFS status, overall survival (OS), OS status, PD-L1 expression), mutational (TMB), and neoantigen data (NetMHCpan and Ancer neoepitope burdens) for each bladder cancer patient.


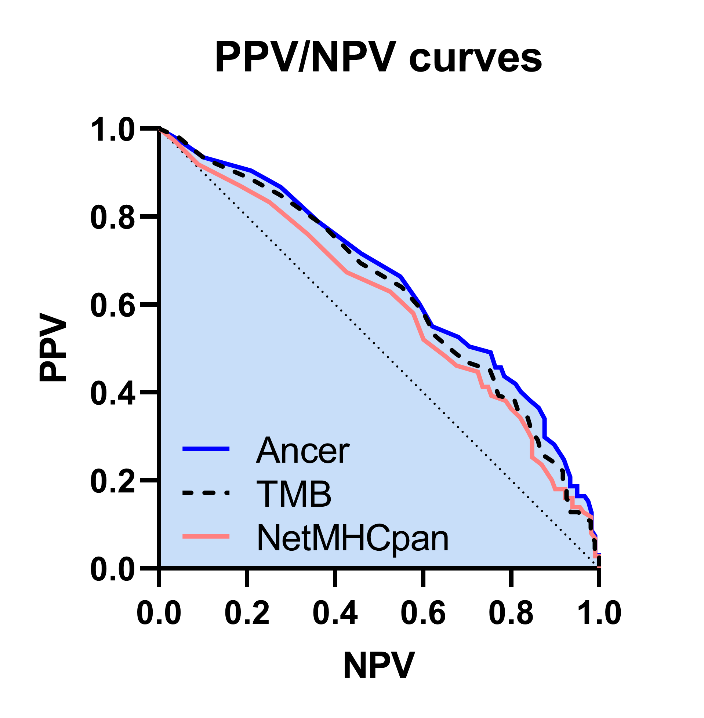


**Supplemental Figure 1. PPV/NPV curves for the TMB, NetMHCpan, and Ancer analyses.**

The TMB, NetMHCpan, and Ancer predictors were used to predict if TCGA BLCA patients survived more or less than a pre-determined amount of time. This analysis was repeated for every 3 months between t=0 and t=14 years. PPVs and NPVs were calculated by comparing the predicted survival status (deceased/alive) with observed overall survival. PPVs and NPVs were plotted for each predictor to calculate Areas Under the Curve (AUC). These curves showcase the improved prediction of patient survival status using Ancer (AUC = 0.6506, blue shaded area) across a continuum of time intervals compared to analyses using TMB (AUC = 0.6270) or NetMHCpan (AUC = 0.5991).
